# Evaluation of a Bi‐Planar Robot Navigation System for Insertion of Cannulated Screws in Femoral Neck Fractures

**DOI:** 10.1111/os.12450

**Published:** 2019-05-23

**Authors:** Meng He, Wei Han, Chun‐peng Zhao, Yong‐gang Su, Li Zhou, Xin‐bao Wu, Jun‐qiang Wang

**Affiliations:** ^1^ Department of Trauma Orthopaedics Beijing Jishuitan Hospital Beijing China

**Keywords:** Femoral neck fractures, Bi‐planar robot navigation system, Cannulated screw fixation

## Abstract

**Objective:**

To evaluate the bi‐planar robot navigation system for insertion of cannulated screws in femoral neck fractures.

**Method:**

Between January 2016 and December 2016, 60 patients with femoral neck fractures were separately treated using percutaneous cannulated screws assisted by the bi‐planar robot navigation system (robot group) and conventional freehand surgery (freehand group). The fluoroscopy time, the number of drilling attempts, and the operation time were recorded during operations; the dispersion and parallelism of the cannulated screws on the posteroanterior and lateral images were measured after operations. Patients were followed up for 12–24 months and the Harris scores and the final results of the two groups were compared.

**Results:**

During bi‐planar robot navigation system‐assisted surgery, the fluoroscopy time for acquisition of images was 2.3 seconds on average, and the time for planning screws during the operation was 2.8 min on average. The average fluoroscopy time during the placement of the guide pin was 5.7 seconds and 14.14 seconds (*P* = 0.00), respectively. The average time of the placement of the cannulated screws was 12.7 min and 19.4 min (*P* = 0.00), respectively, in the robot group and the freehand group. In the robot group, only one guide pin was replaced during the operation, and the average number of adjustments for each guide pin was 2.39 in the freehand group. The screw parallelism and dispersion measured by postoperative imaging in the robot group were significantly superior to those in the freehand group. From postoperative CT it was evident that there were 5 cases of screws exiting the posterior cortex in both groups. During the follow‐up phase, 1 case of femoral head necrosis and 5 cases of femoral neck shortening of more than 10 mm occurred in the robotic navigation group; 3 cases of femoral head necrosis, 1 case of fracture nonunion, and 2 cases of shortening of more than 10 mm occurred in the freehand group. At 18 months after surgery, the average Harris scores of the patients were 85.20 and 83.45, respectively, with no significant difference.

**Conclusion:**

Using bi‐planar robot navigation system‐assisted placement of femoral neck cannulated screws can significantly reduce the time of intraoperative fluoroscopy, drilling attempts, and operation time. The placed screws are superior to the screws placed freehand in relation to parallelism and dispersion. However, it is still necessary for surgeons to have a good reduction of the femoral neck fracture before surgery and to be proficient in the operation of the robot navigation system. In summary, the bi‐planar robot navigation system is an effective assistant instrument for surgery.

## Introduction

Femoral neck fractures are common in the elderly, accounting for 3.58% of total body fractures and 54% of hip fractures. In young people, the incidence is relatively low, with fractures tending to be caused by high‐energy injuries, and accounting for only 2% to 3% of all femoral neck fractures^1^, ^2^. The treatment of femoral neck fractures often results in many complications due to poor reduction, fixation instability, and osteoporosis^3^. According to a meta‐analysis, treatment is associated with high incidences of avascular necrosis of the femoral head (14.3%), nonunion (9.3%), malunion (7.1%), implant failure (9.7%), and surgical site infection (5.1%)^4^. Femoral neck fractures have higher requirements for treatment; otherwise, complications are more likely to occur.

It is believed that precise screw placement can ensure more stable fracture fixation, thus reducing the occurrence of related complications. Some studies indicate that ideal placement of screws allows the reduction of the fracture to show a good reduction in the posteroanterior and lateral images, while reducing the number of drilling attempts can reduce the iatrogenic fractures due to multiple drilling attempts^5^, ^6^, ^7^. The distal screw placement point should be above the smaller trochanter to reduce the risk of subtrochanteric fractures, so the angle of the screw placement should not be too large^8^. It is also reported that, after the screw is placed, the head end of the cannulated screw should be located within 5‐mm below the cartilage of the femoral head; and the cannulated screws should have cortical support within 3 mm of the femoral neck cortex. However, the ideal screw placement brings great difficulty to the operation. During conventional freehand operations, the surgeon needs a wealth of experience and more accurate screw placement can be achieved by proceeding with the operation step by step under fluoroscopy. In recent years, the navigation‐assisted placement of femoral neck screws has emerged as a new technology. Studies have shown that the use of navigation can provide accurate placement of the screws, while reducing the radiation exposure of the surgeon and the number of drilling attempts^9^. However, it only helps the surgeon to see and surgical experience and hand stability remain critical.

The bi‐planar robot system (TINAV) is based on 2D image navigation, can determine the ideal placement of the cannulated screws based on the posteroanterior and lateral images of the hip joint after the reduction, and subsequently provide the placement channels for the screws with its mechanical arm according to the planned route. The simulation surgery experiments show that the bi‐planar robot navigation system‐assisted placement of screws can be more accurate and reduce the number of drilling attempts compared to normal navigation, and does not increase operation time^10^. However, little clinical data has been reported so far.

We used data from 30 cases of robot‐assisted femoral neck cannulated screw placement and 30 cases of freehand placement of cannulated screws collected in 2016. We then comprehensively evaluated the bi‐planar robot navigation system and analyzed strengths and weaknesses. First, we compared the intraoperative fluoroscopy, drilling attempts, and the operation time of the two groups to evaluate its contribution to the operation. Second, after surgery, the parallelism and dispersion of screws in posteroanterior and lateral images were measured. Third, through follow up of the patients, the Harris score of hip joints were mathematical statistics, and related complications were compared. In what follows, we analyze the advantages and related deficiencies of robot‐assisted surgery.

## Materials and Methods

### *General Clinical Data*

We selected 60 patients with femoral neck fractures in 2016 and randomly divided them into two groups. Inclusion criteria: (i) patients with femoral neck fractures; (ii) patients treated with inverted triangle cannulated screws by using the robot navigation system or in traditional method; and (iii) patients aged over 18 years. Exclusion criteria: (i) poor general condition to receive the examinations; (ii) with other lower limb fractures; and (iii) other diseases like cerebral infarction that may affect patient walking. Posteroanterior and lateral X‐ray images and CT were obtained for all patients with femoral neck fractures before surgery, and we recorded the Garden classification of femoral neck fractures. The demographic data of the patients is listed in Table 1. The patients were randomly divided into two groups, and separately treated with robotic‐assisted internal fixation and with conventional freehand fixation with cannulated screws. There were no significant statistical differences in gender, age, cause of injury, time to operation from injury, injury side, and fracture type between the two groups, and they were comparable (*P* > 0.05). We performed a retrospective study.

**Table 1:** Patient information

Information	Robot group	Freehand group
Age (years)	56 (39–82)	56.2 (30–84)
Gender (male/female)	11/19	12/18
**Fracture type (cases)**		
Garden I	8	10
Garden II	5	7
Garden III	14	11
Garden IV	3	2

### *Operative Procedure*

#### *Robot Group*

##### *Navigation Robot Surgery Method*

**Figure 1:**
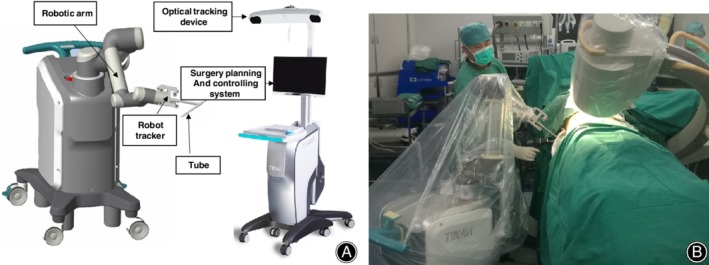
The bi‐planar robot navigation system construction (A) includes a robotic arm, an optical tracking device, and a surgery planning and controlling system. (B) The robot arm provided a guide channel for the surgeon during the operation.

**Figure 2:**
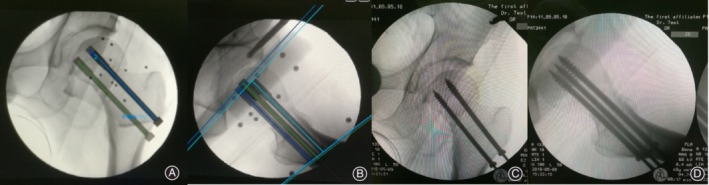
The surgeon could place the screw in the desired position through the workstation of the system (A, B). The robot arm would then provide a guide channel to help the surgeon achieve screw placement in a precise position. From the intraoperative anteroposterior (C) and lateral (D) radiographs, we can see that the screws were placed in the surgeon's planned position.

After anesthesia, all femoral neck fractures were closed reduction. With satisfactory fluoroscopy results, three inverted triangular parallel dispersing cannulated screws were placed using a new bi‐planar navigation robot, TiRobot (TINAVI Medical Technologies,Beijing, China). The navigation robot consists of a multi‐degree‐of‐freedom robotic arm, an optical tracking device, a surgical planning and controlling system, and surgical instruments that help the robot establish spatial coordinates (Fig. 1). The bi‐planar navigation robot needs to place a specific tracking device on the patient's tibia after the fracture is reduced. This optical tracking system is a binocular camera based on infrared light, which can bring the spatial position error to less than 0.3 mm, so that the robot can track the patient in real time^11^. Then a positioning device is connected, appropriate posteroanterior and lateral X‐ray films of the hip joint are captured, and the patient's imaging information is collected by the robot operating system. The operator then plans the screw position on the operating platform. After planning, the operation screen will show whether the screws are parallel, together with the length of the screws (Fig. 2). The robotic arm will then run to the corresponding position according to the planning, and the screw is placed in the channel. The surgeon places the guide pins one by one according to the conventional screw placement method. After achieving satisfactory fluoroscopy results, the cannulated screw is placed.

#### *Freehand Group*

In the conventional freehand fixation group, the screws were inserted with the use of standard c‐arm fluoroscopy after satisfactory closed reduction. Based on anteroposterior and lateral images, the screws were inverted in a satisfactory position.

### *Data Collection and Imaging Measurements*

#### *Intraoperative Related Information*

After fracture reduction and satisfactory results of fluoroscopy were achieved in these two groups of patients, the number of fluoroscopy images during the insertion of screws during operation was recorded. The number of drilling attempts was also recorded. The number of drilling attempts is defined as: each time the needle is repositioned or the angle is readjusted during the insertion process^12^. Finally, the time from the reduction to fully placing the screws was recorded. The robot group also needs to record the time of fluoroscopy when the robot collected the patient's imaging information. After obtaining the image information, the screw position was planned and the operator's planning time was also recorded.

#### *Imaging Measurement after Operation*

After the cannulated screw was placed, anteroposterior and lateral X‐ray images of the hip joint were taken. The parallelism and dispersion of the screws was measured on the plain and lateral films using mimics 19 measurement software. The two tangential points closest to the curvature of the femoral neck were measured, by connecting the points of tangency and measuring the distance between the outer edge of the screws, and the width of the femoral neck. The dispersion percentage is defined as the ratio of the distance between the screws to the width of the femoral neck. On the anteroposterior and lateral images, the angle between the screws and the femoral shaft was determined, and the angle difference between the screw and the femur. θ = (|θ1 – θ2 | + |θ1 –θ3 | + |θ2 –θ3 |)/3^13^°.

#### *Postoperative Patient Follow Up*

The patients in the two groups were followed up for 12–24 months. The fracture healing and related complications were observed. The Harris scores were taken in the two groups.

### *Statistical Analysis*

Data were analyzed descriptively by using means, Student's *t‐*test, and Fisher's exact test. A 5% significance level was applied for all tests (*P* < 0.05). statistical software (IBM SPSS Statistics22; SPSS, Chicago, Illinois, USA) was used for all statistical analyses.

## Results

### *Preparation Time of the Robot System*

Finally, relevant data from the operation and postoperative imaging data were collected. In robot navigation‐assisted surgery, the fluoroscopy time for acquisition of images was 2.3 seconds on average; the time for screws planing during the operation was 2.8 min on average.

### *Intraoperative Fluoroscopic Time*

The average fluoroscopy time during the guide pin placement was 5.7 seconds in the robot group; in the freehand group, the time was 14.14 seconds. Using the robot system can significantly decrease the fluoroscopic time (*P* < 0.01).

### *Number of Drilling Attempt*

Only one guide pin was readjusted in all 30 robot assisted operations. However, in the freehand group, the average number of adjustments of guide pins was 2.39 times. Obviously, the robot system can help surgeons to insert the screw accurately in the ideal position.

### *Total Placement Time of Cannulated Screws*

The average time for the placement of three cannulated screws was 12.7 min in the robot group and 19.4 min in the freehand group. Using the robot system can significantly decrease the operation time (*P* < 0.01).

All intraoperative statistics of the two groups are shown in Table 2.

**Table 2:** Intraoperative data

Groups	Number of patients	Fluoroscopy time for each screw placement (s)	Number of drilling attempt (times)	Total placement time of cannulated screws (min)
Robot navigation group	30	5.65	0.01	12.6
Freehand group	30	14.14	2.39	19.4
Statistics	60	*t* = −9.49, *P* = 0.00	*t* = −12.71, *P* = 0.00	*t* = −4.59, *P* = 0.00

### *Parallelism of Screws*

The anteroposterior screw shaft angle was 1.08° in the robot group versus 1.2° in the freehand group (*P* = 0.437) and the lateral screw shaft was 1.25° in the robot group versus 1.82° in the freehand group (*P* = 0.028). The parallelism of the robotic group screws in the lateral image was significantly better, and there was no significant difference in the parallelism in the anteroposterior image.

### *Dispersion of Screws*

The average anteroposterior dispersion percentage was 65.13% in the robot group versus 58.29% of the femoral neck (*P* < 0.01) in the freehand group; the lateral dispersion percentage was 70.08% versus 61.53% (*P* < 0.01). The screws inserted in the robot group have better dispersion.

Postoperative imaging measurements of the two groups are shown in Table 3.

**Table 3:** Statistics of postoperative image measurements

Groups	Number of patients	Anteroposterior dispersion percentage (%)	Lateral dispersion percentage (%)	Anteroposterior screw shaft angle (°)	Lateral screw shaft angle (°)
Robot navigation group	30	65.13	70.08	1.08	1.25
Freehand group	30	58.29	61.53	1.2	1.82
Statistics	60	*t* = −4.492, *P* = 0.00	*t* = −5.11，*P* = 0.00	*t* = 0.782，*P* = 0.437	*t* = 2.259，*P* = 0.028

### *Follow‐up Results*

During the follow‐up period of 12–24 months, we took anteroposterior and lateral X‐ray images of patients every 1–3 months. After the fracture has healed, we graded the patients’ Harris hip scores. The Harris hip scores of the navigation robot group and the freehand group were 85.20 and 83.45, respectively (*P* > 0.05) with no significant difference.

## Complications

There was 1 case of femoral head necrosis among the 30 patients in the robot navigation group. A total of 5 patients had severe shortening of the legs after fracture healing, and the femoral neck shortened more than 10 mm. The other patients had no obvious shortening with fracture healing. In the freehand group, 3 of 30 patients underwent hip arthroplasty after femoral head necrosis; 1 patient had non‐union and a failed internal fixation; 2 patients had severe fractures after fracture healing, with the femoral neck shortening more than 10 mm.

**Figure 3:**
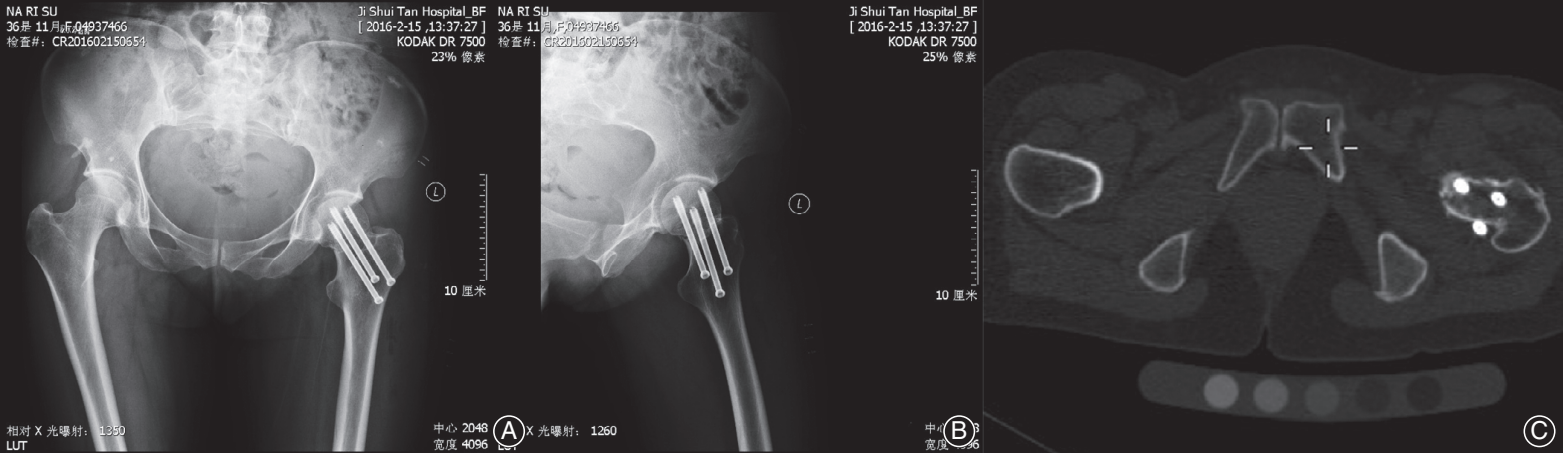
From anteroposterior (A) and lateral (B) X‐ray images, the screws appear to be in the neck of the femur, but, in fact, a screw penetrates the cortex in the posterosuperior of femoral neck (C) in the cross‐section from CT.

Through postoperative CT, we found that both groups had 5 patients with screws exiting the posterior cortex. In both anteroposterior and lateral X‐rays images, the screws appear to be in the neck of the femur but actually penetrate the cortex (Fig. 3).

## Discussion

Computer‐assisted orthopaedic surgery (CAOS) has been gradually recognized as a useful technique over the past few years. CAOS can improve visibility to the surgical field and operational accuracy through navigation systems and robotic devices during surgical procedures^14^. in vitro simulated surgery using cannulated screws to treat femoral neck fractures demonstrated that the CAOS system significantly improved the parallelism of screw placement and the dispersion of the screws in the femoral neck^10^. With the aging of the population, the incidence of femoral neck fractures is increasing, along with complications, so treatment requirements are becoming more stringent. Precise screw placement is believed to provide a more stable fixation. Femoral neck fracture fixation using 2D planar navigation robotic‐assisted cannulated screw placement is undertaken in this study.

### *Intraoperative Data*

In the robot group of 30 patients, all the cannulated screws were placed in an ideal position after reduction of fractures. When using the bi‐planar robot to assist in the placement of the guide pin, only one guide pin was readjusted; the operation time of the cannulated screw insertion was 12.6 min, and the average number of drilling attempts in the freehand group was 2.39 times. The operation time was 19.4 min, and the use of the robot can achieve more precise placement of the guide pin than freehand. In addition, compared with Marcus Christian Müller's use of 3D navigation in 2012, the average operation time was 38 min, the drilling attempts was 3 times, bi‐planar robot could reduce the number of needle adjustments and the operation time^12^. This is due to the screw placement under 3D navigation, which still requires freehand positioning and subsequent adjustment under navigation, which is related to the experience of the operator. The robot navigation system provides more precise and fixed channels for the robot arm. It saves time in surgery and reduces the number of perspectives. At the same time, the risk of iatrogenic fractures is avoided because of the reduction in drilling attempts. Because the guide pins do not need to be adjusted repeatedly, and after the planned screws are placed, the operating platform can provide the length of the screws, which can also help to complete the screw placement and reduce fluoroscopy times.

### *Postoperative Imaging Measurements*

Compared with the freehand group, the robot group had significant advantages in terms of the positive and lateral dispersity and lateral parallelism of the posterior cannulated screws. In this study, the third‐generation robots were used, which have an improved manipulator arm and an optical tracking system that allows minor changes in the procedure to be adjusted in time to make the surgery more accurate. Compared with Meir Liebergall's navigation screw placement study, the angle between the screws in the positive position is 1.34° versus 1.08°, and the lateral position is 1.68° versus 1.25°. The screws placed in the robotic navigation system are more parallel when placed under the navigation^13^. In the degree of dispersion, the robot navigation system has a greater advantage. The ratio of screw holding distance to the femoral neck in the positive position is 65.13%, which is much larger than the degree of dispersion under simple navigation. This is because when the robot‐assisted femoral neck cannulated screw is placed, the robotic arm provides a guide needle insertion channel, and the surgeon can safely place the screws tangent to the femoral neck cortex to obtain the cortical support of the screw. At the same time, because the mechanical arm provides a relatively stable passage, uncontrollable deviation of the guide needle caused by the shaking of the hand during the operation of the operator is avoided, so that the postoperative screw placement is more parallel.

Through postoperative CT we found that both groups had 5 patients with screws exiting the posterior cortex, which may decrease the stability of fixation and injure the blood supply to the femoral head^15^. This may have occurred because the surgeon wanted to place the screws peripherally and close to the femoral neck cortex so that the screws would obtain cortical support and better dispersion. Zhang *et al*.^16^. report that when screws appear close to the cortex on both radiographs, screws may have already perforated the cortex. The plan of where to place the screws is very important, but because of the insufficient information in 2D images, we cannot completely solve this issue at present.

Cortex support has also been shown to be more important in patients with osteoporotic femoral neck fractures. Orbel Filipov's biplane double‐supported screw fixation (BDSF) multi‐angle screw fixation has been demonstrated in biomechanical experiments and postoperative follow up as a possible effective method for the treatment of osteoporotic femoral neck^17^. However, the insertion angle of the screws is difficult to control, and the robot navigation system can better provide the needle guide channel due to the presence of the mechanical arm.

### *Clinical Outcomes*

Postoperative femoral head necrosis and neck shortening after fracture healing occurred in both groups. There was no significant difference in hip Harris score between the two groups. As a unique technology to assist in operations, the navigation robot still needs to rely on the operator's preoperative reduction of the fracture and the planning of the placement of the screws. Therefore, the results of the two groups of patients after final follow‐up are similar. Shortening of the femoral neck of more than 10 mm will affect a patient's function^18^. Shortening may be related to the patient's fracture type, osteoporosis, and screw placement. At present, the robot can only assist in the placement of the screws, and the screw planning depends on the experience of the surgeon.

### *Current Defects of the Robot Navigation System*

There are also shortcomings in the navigation robot system. First, because the navigation robot system needs to collect preoperative images and connect with related instruments and equipment, the preoperative preparation time is long. Second, because the ilium needs to be placed with an optical tracer, it will cause some damage to the patient. The connection between the optical camera and the tracer marker cannot be blocked during the procedure. Third, the spatial error of the bi‐planar navigation robot is 0.3 mm, but in this study, the angle of the worst parallelism of the screw is a poor 2.32°, and the lateral difference is 3.81°. This may be due to the fact that the mechanical arm is too close to the patient and the soft tissue is blocked. Fourth, because the channel provided by the mechanical arm is external, when the guide needle passes through the femoral neck cortical bone and femur, due to the harder bone barrier, the small elastic deformation may cause the guide needle to deflect in the bone, resulting in deviations in the original planning direction, which requires the surgeon to have a wealth of surgical experience and make adjustments through the surgery. Fifth, at present, robot navigation systems rely on the experience of the surgeon to conduct appropriate intraoperative planning.

The navigation robot can help the surgeon with screw planning and in precise needle positioning. However, the reduction of the fracture before surgery is very important. Only after the fracture is completely reduced can the operation achieve better curative effects. At the same time, the plan before the surgeon puts in the screws is also very important. The correct planning of screws is important for a successful operation.

### *Conclusion*

When the bi‐planar robot navigation system is used to assist in femoral neck cannulated screw placement, the time of intraoperative fluoroscopy, drilling attempts, and operation time can be significantly reduced. In the present study, the bi‐planar robot navigation group had better screw parallelism and greater spread of screws. However, it is still necessary for the surgeons to have a good reduction of the femoral neck fracture before surgery and to be proficient in operating the robot navigation system. The bi‐planar robot navigation system is an effective assistant instrument for surgery.
